# Smart Materials Based on DNA Aptamers: Taking Aptasensing to the Next Level

**DOI:** 10.3390/s140203156

**Published:** 2014-02-18

**Authors:** Emily Mastronardi, Amanda Foster, Xueru Zhang, Maria C. DeRosa

**Affiliations:** Department of Chemistry, Carleton University, 1125 Colonel By Drive, Ottawa, ON K1S5B6, Canada; E-Mails: emilymastronardi@gmail.com (E.M.); amandafoster115@gmail.com (A.F.); xueruzhang920@hotmail.com (X.Z.)

**Keywords:** aptamers, hydrogel, gated nanoparticle, nanopore, polyelectrolyte multilayer, Layer-by-Layer, microcapsules, targeted delivery, logic gates

## Abstract

“Smart” materials are an emerging category of multifunctional materials with physical or chemical properties that can be controllably altered in response to an external stimulus. By combining the standard properties of the advanced material with the unique ability to recognize and adapt in response to a change in their environment, these materials are finding applications in areas such as sensing and drug delivery. While the majority of these materials are responsive to physical or chemical changes, a particularly exciting area of research seeks to develop smart materials that are sensitive to specific molecular or biomolecular stimuli. These systems require the integration of a molecular recognition probe specific to the target molecule of interest. The ease of synthesis and labeling, low cost, and stability of DNA aptamers make them uniquely suited to effectively serve as molecular recognition probes in novel smart material systems. This review will highlight current work in the area of aptamer-based smart materials and prospects for their future applications.

## 1. Introduction

“Smart” materials are advanced materials whose physical or chemical properties can change in response to an external stimulus such as temperature, pH, and electric or magnetic fields [1]. Their unique ability to recognize, adapt to, and report on changes in their environment affords these materials unique applications in emerging areas such as sensing and drug delivery. For example, electrochromic, thermochromic, and photochromic materials change color in response to a change in applied voltage, temperature, or light, respectively [2–5]. Piezoelectric smart materials have been described that can sense and scavenge vibrational energy and respond by generating electrical energy to power devices [6,7]. Shape memory materials have been developed that can change their shape in response to temperature, stress, or magnetic field changes [8,9]. Smart material systems are finding diverse applications in sensing, molecular electronics, and controlled delivery.

A particularly exciting area of research seeks to develop smart materials that are sensitive to specific molecular or biomolecular stimuli. These systems integrate a molecular recognition probe within the material structure that is specific to the target molecule of interest. In comparison to other molecular recognition probes such as antibodies or molecularly imprinted polymers (MIPs), aptamer technology is particularly compatible with smart material systems [10,11]. Aptamers are synthetic, nucleic-acid based receptors that fold into unique three-dimensional structures capable of binding tightly and selectively to a target of interest [12,13]. Aptamers have been developed for a wide variety of molecules that could serve as triggers for smart materials, from small molecules such as amino acids to very complex targets such as bacteria, and whole cells [14–17]. The relatively low cost of production, ease of synthesis and labelling, as well as the stability of DNA aptamers make them uniquely suited to effectively serve as molecular recognition probes in novel smart material systems. Furthermore, unlike other affinity ligands such as antibodies or MIPs, the binding functionality of nucleic acid aptamers can be regulated through hybridization with their complementary sequences. This provides an extra layer of control to smart materials based on aptamers.

This short review will summarize the latest developments in the area of aptamer-based smart materials. For the purpose of this review, these systems will be defined as materials that demonstrate target-molecule-derived functionality as a result of aptamer inclusion into a material system (polymer, nanomaterial, *etc.*). These hybrid materials do more than aptasensing, or simple reporting on the presence of a target. The presence of the target is used as a stimulus to trigger further changes to the system such as the degradation of the material or release of a molecular payload. The examples describe the marriage of aptamer technology with material science to yield multifunctional materials with advanced, tunable properties.

## 2. Aptamer-Based Hydrogels

**Figure 1.**
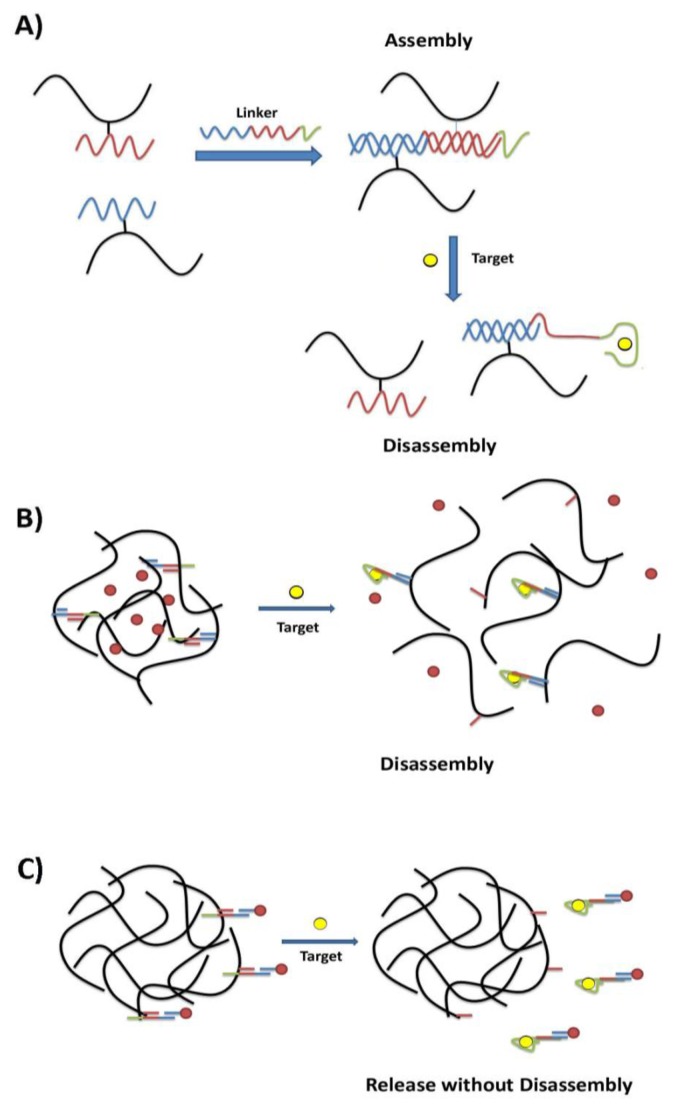
Smart aptamer-hydrogel systems for controlled release. (**A**) Initial assembly of the gel is caused by the hybridization of a DNA linker strand (blue-red-green) with complementary DNA segments tethered to the polymer subunits (blue and red). The linker strand contains an aptamer domain (green) which preferentially binds to the target molecule (yellow sphere), leading to disassembly of the gel. (**B**) This system could be applied as a smart material for the target-responsive release of a molecular payload, e.g., gold nanoparticles. (**C**) A second strategy for controlled release where the payload is attached to the hydrogel by an aptamer-containing linker strand. Target binding and payload release do not require disassembly of the gel. (modified from references [20,21]).

Hydrogels are one class of materials that displays stimuli-responsive changes in their structural network [18]. Hydrogels are composed of a crosslinked hydrophilic polymer network that readily takes up water. The amount of swelling is influenced by conditions such as pH, ionic strength, temperature, light, electric field, and solvent choice and these parameters are often exploited in stimuli-responsive hydrogels [19]. In recent work, DNA aptamers have been used to translate the recognition of a specific analyte into the controlled phase transitions of a hydrogel material. Tan and coworkers designed a hydrogel system where the polymer gel was initially crosslinked by the hybridization of DNA tethered to the polymer subunits with a rationally designed DNA linker strand [20]. (See Figure 1A) The linker strand was designed to have an aptamer domain (ATP aptamer) in addition to segments partially complementary to those sequences conjugated to the polymer subunits. As a result, in the presence of the aptamer's target, preferential binding to the molecular stimulus leads to the break-up of the crosslinks and the disassembly of the gel. This disassembly event could therefore be exploited to achieve target-driven payload delivery. (See Figure 1B)

The Tan group showed that gold-nanoparticles (AuNPs) loaded in this hybrid hydrogel could be released in the presence of adenosine. A thrombin aptamer-based hydrogel was also prepared, however disassembly occurred with much slower kinetics than the adenosine system. It was postulated that the larger-size of thrombin as compared to adenosine would decrease the rate of diffusion into the hydrogel, and therefore slow disassembly speed. Thus, disassembly of the smart hydrogel could be tuned through the choice of aptamer-target system. The same group later created a colorimetric assay using amylase enzyme encapsulated in a cocaine aptamer-based hybrid hydrogel [22]. The colorimetric assay was designed exploiting the color change that occurs when iodine is in the presence of the polysaccharide amylose (yellow to indigo). However, if the amylose is digested (via the amylase enzyme) the dark blue color will disappear leaving a colorless solution. Exposure of the hydrogel to cocaine led to the release of amylase from the disassembled material and the loss of the characteristic indigo color. The time required for complete disassembly (and therefore the stability of the hydrogel) could be tuned depending on the crosslink density of the hydrogel. The system also showed specificity towards cocaine as exposure to benzoylecgonine and ecgonine did not trigger enzyme release. This system serves as a model for not only cocaine sensing but also controlled enzyme delivery, which could be applied to other scenarios such as bioremediation and drug delivery.

An alternate approach to the development of smart hydrogels has the payload tethered to the hydrogel by a DNA linker containing the aptamer sequence (Figure 1C). The difference in this case is that aptamer binding only disrupts the linker attachment to the hydrogel but does not affect hydrogel crosslinking or lead to total hydrogel disassembly. Liu and coworkers developed a target-responsive hydrogel where gold nanoparticles, as the model cargo, were bound to the hydrogel by the adenosine aptamer [21]. In the presence of the target, the aptamer would preferentially bind adenosine over the hydrogel, releasing the gold nanoparticles from the hydrogel surface. Release was seen only with targets known to bind the aptamer, confirming the specificity of the approach. The rate of cargo release could be tuned based on ionic strength of the solution as well as temperature, and the system was very effective under physiological conditions. The hydrogel could also be dried and rehydrated, showing tighter cargo binding and slowed release. When rhodamine-labeled DOPC liposomes were used as a cargo in place of the gold nanoparticles, the same release properties were found, confirming the generality of this approach.

The challenge of developing a system able to control the release of multiple protein drugs at distinct time points was examined using a dual aptamer-hydrogel composite system [23,24]. Here, the vascular endothelial growth factor (VEGF) and platelet-derived growth factor BB (PDGF-BB) aptamers were used to functionalize streptavidin-coated microparticles that were physically incorporated into an agarose hydrogel network. Once loaded with the two protein payloads, complementary sequences could be used as triggers to release the specific payload at defined time points. After demonstrating that the composites could retain the proteins, the composites were incubated with the complementary trigger strands. It was found that the daily release rate of VEGF after triggering with an optimized VEGF complementary sequence increased from 1% to ∼14%, whereas PDGF-BB release was not affected. Moreover, the daily release rate of PDGF-BB after triggering with the optimum PDGF-BB complementary sequence increased from 0.5% to ∼6%, whereas the VEGF release was unaffected. This smart material represents a promising platform for the controlled release of multiple payloads with adjustable release rates and times.

**Figure 2.**
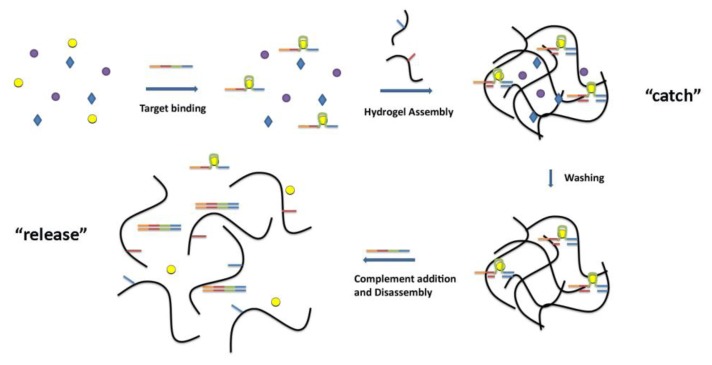
Catch and release smart materials: A crosslinker strand (orange-red-green-blue) containing an aptamer domain (green) for the target of interest (yellow circles) binds to the target and assembles the hydrogel through complementary interactions with the DNA-polymer conjugates (red and blue). After washing to remove any nonspecifically-held species, the purified target can be “released” through disassembly of the gel. This is accomplished by introducing a fully complementary strand that can unzip the crosslinker strand from the DNA-polymer conjugates starting at a single-stranded toe-hold sequence (orange). (modified from reference [25]).

Hydrogel systems could also be adapted as smart materials for “catch and release” [25]. (Figure 2) Mi and coworkers presented a method to separate a specific target from a mixed system using DNA aptamers incorporated into a reversible hydrogel system. In this system, a crosslinker strand was prepared containing three key domains: (1) an aptamer sequence for binding the target of interest, (2) sequences that are complementary to the DNA-polyacrylamide conjugates for assembly of the hydrogel, and (3) a “toehold” domain to initiate hydrogel disassembly. The crosslinker strand was incubated in a complex mixture containing the target. After target binding, the polyacrylamide-DNA conjugates were added to assemble the hydrogel. Washing removed any nonspecifically-held species, and the purified target was “released” through disassembly of the gel, by introducing a strand that is complementary to all domains on the crosslinker. This strand unzipped the crosslinker strand from the DNA-polymer conjugates starting at the single- stranded toe-hold sequence. Using this catch and release approach, the authors were able to separate ATP from a pool of ATP and GTP, as well as α-thrombin from a pool of α-thrombin and bovine serum albumin. This example shows that aptamer-based smart materials may find applications in molecular separations for environmental and medical applications.

**Figure 3.**
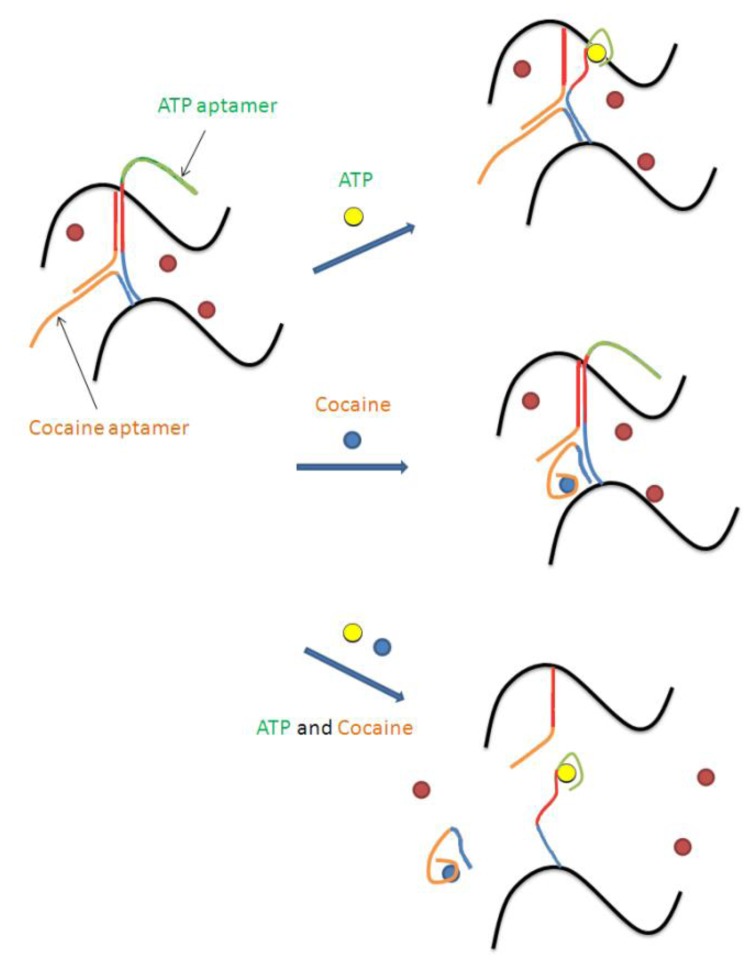
Logic “AND” gate using an aptamer-based smart material. A hydrogel is assembled using three components: two DNA-polymer conjugates and a crosslinker strand. One DNA-polymer conjugate is prepared from a sequence (blue-red-green) that contains the ATP aptamer (green) and is complementary to the other DNA-polymer conjugate (red) as well to the crosslinker (blue). The second DNA-polymer conjugate is prepared from a sequence (red-orange) that is complementary to the cocaine aptamer (orange) and complementary the other DNA-polymer conjugate (red). The crosslinking of these two polymer conjugates is further strengthened by a DNA sequence (orange-blue) that contains the cocaine aptamer (orange) and is complementary to the first DNA-polymer conjugate (blue). In the presence of either cocaine or ATP (top arrow and middle arrow) the hydrogel remains assembled. However, in the presence of cocaine and ATP (bottom arrow) the hydrogel is no longer crosslinked and can disassemble. (modified from reference [29]).

Logic gates are another application for aptamer-based smart materials. Aptamer-based logic gates that respond to multiple targets, or to a target as well as another molecular stimulus (e.g., pH), have been described by several groups [26–28]. Tan and coworkers were able to exploit the combination of aptamers and hydrogels to create two colorimetric logic gates (“AND” and “OR”) using the target binding abilities of aptamers to control hydrogel disassembly [29]. The cocaine and ATP aptamers were used in this study. The dispersal of the red AuNPs was used to assess the state of the hydrogel; confined indicated a gel state (“FALSE”), and dispersed indicated a solution state (“TRUE”). The “AND” gate was represented by the situation where the output was “true” only if both inputs were present. In this model, the inputs were ATP and cocaine. To achieve the hydrogel that would only disassemble in the presence of both targets, sequences were designed such that they could form a Y-shaped structure, with each strand containing two half-complementary domains for the other two strands (see Figure 3).

In the presence of only one target, the respective aptamer segment dissociated from the three strand complex, however enough inter-polymer strand interactions were still maintained to keep the hydrogel together. It was only when both targets were present that the hydrogel reverted to a solution state. Analogs of both targets, such as GTP or benzoylecgonine, were unable to initiate hydrogel disassembly, thanks to the specificity of the two aptamers. Similarly, an “OR” gate was assembled such that the interaction of either aptamer with its respective target led the hydrogel to revert to the solution state. Again, this change could not be achieved using analogs of the targets. This work demonstrates the potential of the aptamer-hydrogel smart materials as a controlled release system with possible applications in biosensors, nanomechanical devices, molecular electronics, and drug delivery devices.

## 3. Aptamer-Gated Nanoporous Surfaces and Nanoparticles

Porous substrates such as nanoparticles or nanostructured surfaces can serve as an excellent foundation for smart materials. Synthetic nanopores are of great interest for several applications, including as potential mimics of biological gated ion channels. Typically, controlled transport through these synthetic pores is achieved by integrating polymers responsive to chemical or physical stimuli. Aptamer-target binding as a means of control of transport through these nanopores has also recently been investigated. Recently, 20 and 65 nm radii glass nanopores were gated by covalently attaching cocaine-binding aptamers to their surfaces [30]. Using cyclic voltammetry of a redox probe, ferrocene dimethanol, the authors were able to demonstrate that aptamer binding to cocaine triggered a conformational change in the aptamer that allowed the orifice of the nanopore to be reversibly switched from a more blocked to a more opened state.

**Figure 4.**
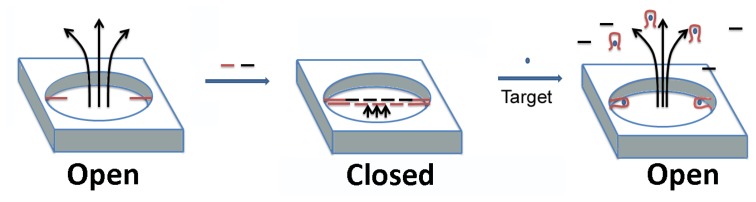
An aptamer-gated nanopore. DNA strands (red) based on the ATP aptamer act as capture probes on an alumina channel wall. Introduction of linking strands and more ATP aptamers (black and red, respectively) lead to the formation of long concatamers through DNA hybridization. These long DNA duplexes block the transport of any ions through the channel. Upon addition of ATP, binding of the aptamer with the target causes the long concatamer to disassemble, opening up the pore and allowing ion transport. (modified from reference [31]).

Jiang *et al* have used the ATP aptamer to develop a smart gated nanopore/nanofluidic channel system with a high ON-OFF ratio and a very good electrical seal (∼GΩ) in the channel's closed state [31]. In this system, a DNA strand acting as a capture probe was immobilized on an alumina channel wall, which hybridized with two other probe DNAs to form long concatamers (see Figure 4). These long DNA strands blocked the transport of any ions through the channel. One of the two probe DNAs as well as the capture probe were ATP aptamers. Upon addition of ATP, the long concatamer “DNA sandwich” disassembled, allowing ion transport. The authors developed this device further into a logic gate, performing the first example of a logical implication operation in a nanofluidic.

**Figure 5.**
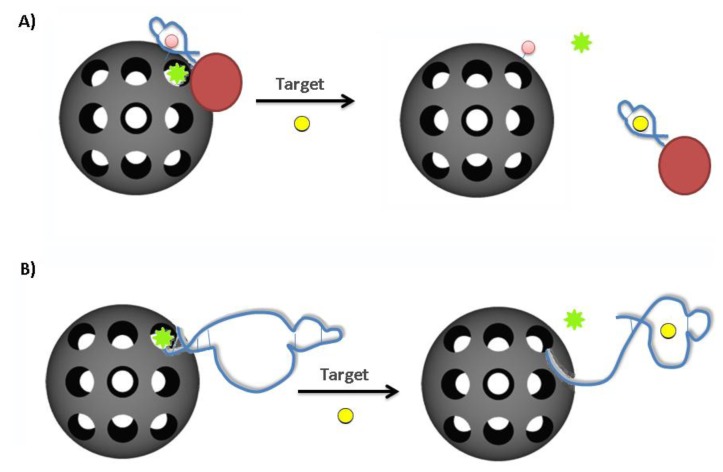
Aptamer-gated nanoparticle systems. (**A**) One-time release system. The pores of a silica nanoparticle are modified with an adenosine derivative (pink sphere) and blocked with gold nanoparticles that are decorated with the ATP aptamer. The presence of ATP in solution competitively displaces the nanoparticles, allowing for the release of the payload (green stars). (**B**) Reversible release system. The conformational change that takes place within the ATP aptamer upon target binding is exploited for payload release. In the unbound state (hairpin), the aptamer effectively blocks the pore opening. Upon target binding, the shape of the aptamer-target complex is less sterically bulky at the pore surface, leading to payload release. (modified from references [33,34]).

Gated nanoparticles typically make use of a mesoporous nanoparticle and a stimuli-responsive, bulky stopper molecule which can effectively block the pore. In these systems, the nanoparticle will serve to protect and transport a given payload and the “gate” will only open to release the payload upon proper stimulus [32]. This has been achieved previously using antibodies and enzymes and recently aptamer-based systems have also been receiving significant attention [32]. Bioresponsive mesoporous silica nanoparticles have been prepared by capping the pore openings with gold nanoparticles (AuNPs) decorated with ATP aptamers [33] (Figure 5A). An adenosine derivative immobilized on the outer surface of the silica NPs provided a binding site for the ATP-aptamer-coated AuNPs. The AuNPs blocked the pores of the silica nanoparticles, preventing the release of a pre-loaded fluorescein dye payload. The addition of free ATP led to a competitive displacement of the ATP-aptamer-coated AuNPs, uncapping the silica pores and allowing for the release of the dye payload. The rate of dye release was dependent on the concentration of ATP and it could not be triggered by target analogs (GTP, CTP, UTP). This system represents an option for a one-time “snap-top” release mechanism exploiting aptamer-target binding.

A reversible gated system has also been devised exploiting conformational changes that can occur within an aptamer structure upon target binding [34]. (Figure 5B) In this example, the structural change of the ATP aptamer system (from a hairpin to an aptamer-target complex) was explored for gating the pores of mesoporous silica nanoparticles. Immobilization of ATP aptamers (in their target-free hairpin structure) to the nanoparticle surface effectively blocked leakage of a fluorescein payload (15 ± 5% leakage). Upon ATP binding, the structure switched leaving a less sterically bulky single-stranded sequence at the pore opening, allowing the fluorescein to be released. The addition of 1mM ATP caused 83 ± 4% of the sequestered fluorescein to be released after 2 h. The gating function of the aptamer was shown to be completely reversible with sequential removal and re-addition of ATP. Specificity of the gating was confirmed as payload release could be not triggered using GTP. In addition to this, the system showed similar efficacy in cell culture medium as in PBS buffer.

**Figure 6.**
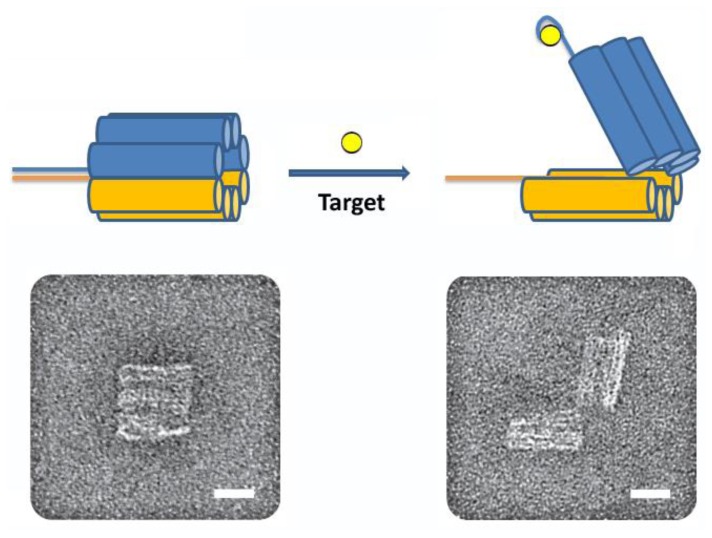
Aptamer-gated DNA nanorobot. Top: Schematic of the nanorobot. A DNA origami-based hexagonal barrel is held together at the front by staples modified with DNA-aptamer-based locks. The robot is opened by target displacement of the aptamer locks. Bottom: TEM images of robots in closed and open conformations (Scale bars = 20 nm) (modified from reference [35]).

In a particularly innovative and exciting example, Church and coworkers created an aptamer-gated particle made completely of DNA [35]. This “nanorobot” was a hexagonal DNA barrel-shaped particle, prepared by DNA origami, attached by single-stranded hinges at the back and two DNA aptamer-complement duplexes as locks in the front (see Figure 6).

These aptamer locks open in response to their target; in this study the authors used an aptamer selected for PDGF and two aptamers selected for CCRF-CEM cells (T-cell acute lymphoblastic leukemia). The authors loaded the nanorobot with fluorescently-labeled antibody fragments that would bind human leukocyte antigen. Upon mixing with several cell types and detecting fluorescence using flow cytometry, the antibody fragments inside the nanorobot were found to only label the cells with the proper combination of aptamer targets or “keys”. By using two different aptamer sequences to lock the nanorobot, the authors created an “AND” gate, and found that the nanorobot was able to discriminate between cell types. The authors also found that the nanorobot was able to induce changes in cell behavior by varying its cargo and targets. For example, the PDGF aptamer nanorobots loaded with a combination of antibodies to human CD33 and human CDw328 Fab' fragments, which have been shown to induce growth arrest in leukemic cells, were able to induce cell-arrest in NKL cells. This combination of DNA origami, aptamer, and antibody technology demonstrates the promise of exclusively biomolecular smart materials, in particular for drug delivery and therapeutics.

## 4. Aptamer-Polyelectrolyte Films and Microcapsules

**Figure 7.**
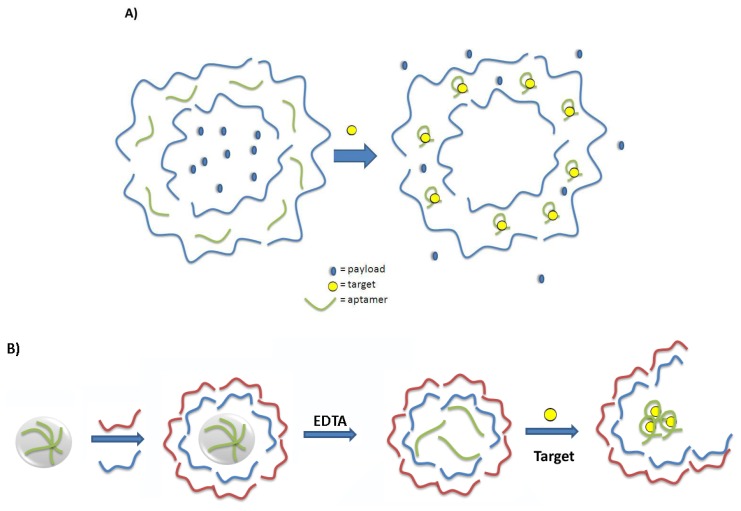
Model smart aptamer-polyelectrolyte microcapsule systems. (**A**) Target (yellow circles) binding to the aptamer (green) within the polyelectrolyte walls of the microcapsule (blue) leads to a change in permeability and release of the microcapsule's payload (blue ovals). (**B**) Aptamers (green) are doped within the sacrificial spherical templates upon which polyelectrolytes (red and blue) are deposited. Upon dissolution of the template, the aptamers support the structure of the polyelectrolyte microcapsule. Target binding (yellow ovals) compromises the structural integrity of the microcapsule and it ruptures. (modified from references [40,42]).

Another popular method for the preparation of smart materials is the Layer-by-Layer (LbL) technique. This technique is based on the sequential adsorption of oppositely charged molecules onto a charged substrate [36]. The nature of the assembly process leads to precise, nanoscale control of film thickness and composition through the appropriate choice of the components and the number of layers, allowing for these films to be tailored to their specific application. Methods to change the permeability or initiate the destruction of these multilayered polyelectrolyte films or microcapsules are generating increased interest in the development of smart materials for controlled release of a molecular payload [37]. There has been more recent attention on designing systems whereby the detection of a target molecule will lead to changes in the polyelectrolyte film and concomitant release of payload. As aptamers are negatively charged biopolymers, their incorporation into polyelectrolyte films as molecular recognition elements has been explored. The successful incorporation of a DNA aptamer into a multilayered polyelectrolyte thin film was first reported in 2009 [38]. It was found that the matrix was flexible enough to permit a model aptamer to fold into its active conformation and to bind the target strongly and specifically, confirming that an aptamer can confer its affinity and specificity for its cognate target to the nanoscale polyelectrolyte film. The targeting ability of aptamers has been used to direct the delivery and internalization of stimuli-responsive polyelectrolyte-based microcapsules [39]. Aptamers have also been incorporated into the walls of hollow polyelectrolyte microcapsules in order to gauge the effect of aptamer-target binding on the permeability of the capsule walls [40]. In this proof-of-concept experiment, the diffusion of the dye sulforhodamine B through the walls of a series of polyelectrolyte microcapsules was monitored using Fluorescence Recovery After Photobleaching (FRAP). In microcapsules containing the sulforhodamine B aptamer within the multilayers, the diffusion coefficient for the dye was nearly an order of magnitude greater than either microcapsules containing a random DNA oligonucleotide, or those comprised of synthetic polyelectrolytes alone. Although the mechanism behind this change in permeability has yet to be confirmed, aptamer-target binding and any associated change in aptamer conformation or volume may lead to the creation of pores within the coating. These pores, in turn, could increase the permeability of the microcapsule wall. This work furthers the possibilities for use of aptamers in smart-responsive coatings for controlled release (Figure 7A) and for innovative sensing platforms [41].

Aptamer loading within the core of polyelectrolyte microcapsules, rather than in the walls, is a second strategy that has been investigated for the preparation of responsive microcapsules (Figure 7B) [42]. In recent work, the sulforhodamine B aptamer was doped into a spherical CaCO_3_ template upon which polyelectrolyte films were deposited. Dissolution of the template left the aptamer sequences encapsulated within the microcapsule core and they were postulated to help serve as an internal scaffold for the microcapsule walls. When the microcapsules were incubated with sulforhodamine B over several days, aptamer-binding triggered the slow collapse and rupture of the microcapsules. Incubation in buffer alone or with tetramethylrosamine (a dye of similar structure but not known to bind the sulforhodamine B aptamer) did not lead to the rupture of the microcapsules.

In these aptamer-polyelectrolyte systems, one can envision applications where these microcapsules control the release of a drug, for example, through aptamer binding to a relevant biomarker or metabolite. In the current system, small molecules able to diffuse into the bulk of the polyelectrolyte coating to bind with the embedded aptamer would be the ideal “triggers” for the release of the capsule's payload.

## 5. Outlook

The examples reported here demonstrate the rich diversity of approaches and applications for aptamer-based smart materials. Future work in this area should focus on moving away from “proof of concept” and into systems that are truly pertinent to real-world applications. With a few exceptions, many of the reports outlined here focus on model aptamers such as those for ATP, sulforhodamine B, and thrombin. These smart materials have the potential to be programmed to respond to a nearly limitless variety of targets, providing universal platforms for a range of applications. To establish the universality of these systems, however, would require mining of the existing plethora of aptamer systems [43] to find relevant and feasible systems as well as the discovery of new aptamers for relevant small molecules, proteins, cells, *etc.* Furthermore, new material systems should also be explored for aptamer incorporation. For example, stimuli–responsive self-immolative polymers are an exciting new class of materials that undergo a stimulus-triggered head-to-tail depolymerisation, often leading to the release of small molecules [44]. If this depolymerisation could be triggered by an aptamer-target binding event, these smart materials could find applications in a wider range of delivery scenarios.

Systems achieving a combination of aptamer targeting and aptamer gating could be explored to yield exquisitely selective smart materials. For example, aptamer-mesoporous silica nanoparticle conjugates have recently been prepared for imaging and drug delivery to breast cancer cells [45]. Here, the aptamer is used solely for its ability to localize the nanoparticle in the tissue of interest. Developing systems that also incorporate gating of the pores could provide two mechanisms to improve the specificity of the delivery system. Dual labeled hydrogel systems whereby one aptamer is used for targeting while the other is employed for crosslinking could also yield some promising new delivery systems. Acoustic phase change droplets or microbubbles are another promising new class of materials deserving further investigation. These microbubbles are typically based on perfluorocarbon-filled, micron-sized lipid shells [46]. Aptamer-coated microbubbles have been developed as ultrasound theranostic agents [47]. A smart aptamer-based microbubble system was recently designed to generate ultrasound signal only in response to the target molecule, thrombin [48,49]. The aptamer serves as more than just a targeting ligand but is also responsible for the microbubble activation, opening up many new possibilities for detection, targeted delivery and treatment.

Finally, new applications for smart aptamer-based materials are worthy of exploration. For example, a recent report by Wang and coworkers used aptamer-functionalized hydrogels to act as binding sites for different cell types, in an effort towards developing an artificial extracellular matrix [50]. The cell-density rose from 5 cells/mm^2^ on a control hydrogel to approximately 850 cells/mm^2^ on the aptamer-functionalized hydrogel, all while causing minimal cell death. The complementary oligonucleotide strands were used to remove the cells, and effectively did so when used in a 1:1 ratio with the aptamer strands. One could imagine moving this a step further to develop smart surfaces for cell growth. The binding of a specific cell type through a cell-surface biomarker to an aptamer on the hydrogel could also trigger the release of required nutrients, or set off the key cell signaling cascades that could aid cell growth.

## 6. Conclusions

The fusion of aptamer technology with material science has yielded smart materials with tunable properties for a range of sensing, molecular electronics, and delivery applications. These hybrid materials take aptasensing to the next level by using the presence of a target molecule as a stimulus to trigger further changes to the system. Three main families of aptamer-based smart materials were presented here: hydrogels, gated nanopores/nanoparticles, and polyelectrolyte films and microcapsules. These systems have successfully demonstrated the feasibility of conferring aptamer-based targeting to a range of materials. Through the combination of further aptamer discovery, new material exploration, and novel application development, these smart materials have the potential to move out of the laboratory and into real-world applications.
